# Surface Stabilization of the Cubic Phase in Lithium Alloyed Double Perovskite Nanocrystals

**DOI:** 10.1021/acs.chemmater.5c02730

**Published:** 2026-03-31

**Authors:** Saar Shaek, Offir Zachs-Maishlos, Lotte Kortstee, Rachel Lifer, Emma H. Massasa, Georgy Dosovitskiy, Yaron Kauffmann, Boaz Pokroy, Ivano E. Castelli, Yehonadav Bekenstein

**Affiliations:** † Department of Materials Science and Engineering, 26747 Technion − Israel Institute of Technology, 32000 Haifa, Israel; ‡ The Solid-State Institute, Technion − Israel Institute of Technology, 3200003 Haifa, Israel; § The Resnick Sustainability Center for Catalysis, Technion − Israel Institute of Technology, 3200003 Haifa, Israel; ∥ Department of Energy Conversion and Storage (DTU Energy), 5205 Technical University of Denmark, Agnes Nielsens Vej 301, DK-2800 Kongens Lyngby, Denmark

## Abstract

The high surface-to-volume ratio in nanocrystals (NCs) enables surface energy effects that stabilize phases that are otherwise unstable in a bulk state. Double perovskites (DP) containing lithium show exactly this effect. Bulk Cs_2_LiInCl_6_ adopts a triclinic structure; however, we show here that the cubic phase can be stabilized in a nanocrystalline form at a wide range of lithium–sodium alloyed compositions. Density functional theory (DFT) calculations support this finding. Although bulk formation energy favors the triclinic structure for Li-rich compositions, the cubic phase becomes stable when the surface energy of the {100} facet is significant due to the large surface-to-volume ratio in nanocrystals. The importance of this Li–Na alloying is seen in the change in the physical properties, apparent in the controllable blue shift of the emission with increased Li content. This advantageous effect, which is also observed for Li-cation exchange in presynthesized colloidal nanocrystals, is overshadowed by a competing phase that we identify as an orthorhombic hydrate phase, Cs_2_InCl_5_·H_2_O. We characterize its emergence and propose strategies to mitigate its impact.

## Graphical abstract

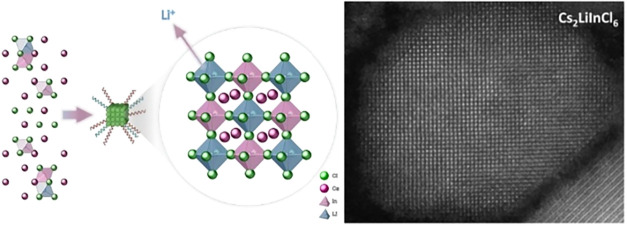

## Introduction

Lead halide perovskites (LHP) emerged in the past decade as promising optoelectronic materials;^1–9^ particularly, inorganic nanocrystals (NCs) with a general formula CsPbX_3_ (X is a halide) gained interest due to notably bright emission and tunable properties.^4,5,10,11^ However, lead toxicity motivated the search for lead-free alternatives, which resulted in the rise of double perovskites (DP) with an elpasolite structure (Cs_2_B^+^B^3+^X_6_).^12–17^ They inherit a similar structural pattern as LHPs, but due to different electronic properties, including an indirect band gap or symmetry-forbidden transitions, they demonstrate poor optical performance.^18,19^ Alloying and doping may break crystal symmetry and allow restricted optical transitions, increasing photoluminescence quantum yield (PLQY) and tuning emission wavelength in bulk and nanomaterials.^20–24^

Recently, control of the emission in Cs_2_AgInCl_6_ DP nanocrystals was demonstrated by inducing a lattice strain via doping them with B^+^ and B^3+^ site cations of different sizes.^24^ Lithium, being the smallest available monovalent metal cation, is of particular interest for studying its effects on DP lattice strain and spectroscopy. While early works on Li-based metal-halide structures were published in 1978 and 1989,^25,26^ Ag, Na, and K were the most common cations used for the B^+^ site during the recent development of the halide double perovskite field.^27–29^ Li-based elpasolites first received attention as neutron-sensitive scintillators.^30,31^ Several experimental attempts to use Li in LHPs were performed in the past decade for intended applications in energy storage, photovoltaics, and light emission,^32–35^ as well as several density functional theory (DFT) calculations of their stability and electronic structure.^35–39^ DPs hosting Li in the B^+^ site with (A_2_Li^+^B^3+^X_6_) have only regained interest in the past year, whereas nanoparticles of these materials have never been studied.^40–44^

Here, we study Cs_2_LiInCl_6_ nanocrystals using an alloying approach to highlight the role of the surface in their stabilization. Bulk Cs_2_LiInCl_6_ is stable at room temperature with trigonal symmetry. However, colloidal nanocrystals of the same composition are stabilized as a double perovskite with cubic symmetry (see Figure 1a,b).^25,26^ We present high-symmetry phases of Li-based double perovskite nanocrystals stabilized at room temperature. We added 10 atom % of Sb, found as the optimum concentration for enhanced emission properties for the Cs_2_Na­(In,Sb)­Cl_6_ NCs.^24^ Previous works demonstrate that [SbCl_6_] octahedra serve as recombination centers, enabling the optical properties of self-trapped excitons.^22,23^

**Figure 1:**
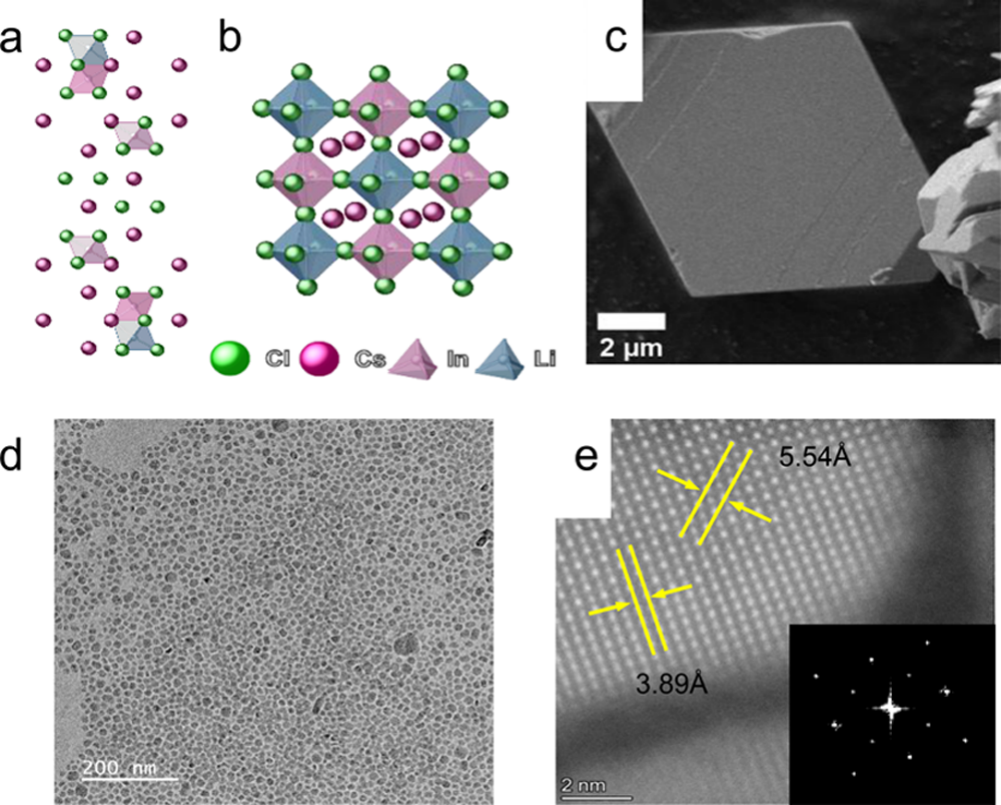
Cs_2_LiInCl_6_ structure model of the (a) trigonal^25^ and (b) cubic DP phase (here and below, all structure models are illustrated using CrystalMaker software).^57^ (c) Scanning electron microscopy (SEM) micrograph of Cs_2_LiInCl_6_ bulk microcrystal. The micrograph was acquired using secondary electron detection mode. (d) Transmission electron microscopy (TEM) micrograph of Cs_2_LiInCl_6_ nanocrystals. (e) High-angle annular dark-field scanning transmission electron microscopy (HAADF-STEM) micrograph ([100] zone axis) with lattice *d*-spacing and fast Fourier transform (FFT) of Cs_2_LiInCl_6_ nanoparticle, confirming the cubic symmetry.

Furthermore, Li alloying enables tuning of the emission wavelength, shifting the emission peak to 440 nm. Analysis of the HAADF-STEM images reveals that the nanocrystals containing both Na and Li ions are the most strained. Nevertheless, we demonstrate that Na–Li ion exchange occurs in the nanoparticles, opening the way to sensing applications.

## Experimental Section

### Sample Fabrication Procedures

#### Materials

Antimony­(III) acetate (99.99%, Sigma-Aldrich), benzoyl chloride (99.9%, Alfa Aesar), lithium acetate (99.995%, Sigma-Aldrich), cesium carbonate (99%, Sigma-Aldrich), hexane (97.0%, Sigma-Aldrich), indium­(III) acetate (99.99%, Sigma-Aldrich), octadecene (ODE) (90%, Sigma-Aldrich), oleic acid (90%, Sigma-Aldrich), oleylamine (≥98%, Sigma-Aldrich), sodium acetate (≥99.9%, Sigma-Aldrich), cesium chloride (≥99.5%, Sigma-Aldrich), and hydrochloric acid (37%, Sigma-Aldrich). All chemicals were used as purchased, with no further purifications.

#### Synthesis of 0.5 M Cs-Oleate and 0.25 M In-Oleate Precursors

The Cs-oleate and In-oleate solutions were prepared following the approach of Locardi et al.^21,45^ 1.63 g (5 mmol) of Cs_2_CO_3_ or 1.4598 g (5 mmol) of In­(Ac)_3_, 20 mL (63.37 mmol) of oleic acid, and a stirring bar were inserted into a 50 mL three-necked round-bottom flask. The flask was plugged into a Schlenk line and degassed under vacuum and at 100 °C for 30 min or until there were no bubbles. Then, the temperature was raised to 150 °C under nitrogen and stirred for 3 h. The product was a clear yellow solution.

#### Synthesis of 0.25 M Sb-Oleate, 0.5 M Li-Oleate, and 0.5 M Na-Oleate Precursors

0.7472 g (2.5 mmol) portion of Sb­(Ac)_3_ or 0.3299 g (5 mmol) of Li­(Ac) or 0.4102 g (5 mmol) of Na­(Ac), 10 mL (31.7 mmol) of oleic acid, and a stirring bar were inserted into a 20 mL glass vial. The reaction was stirred for 45 min at 90 °C in an open-air environment. The Sb-oleate and Na-oleate products were slightly yellow, clear solutions. The In-oleate product was a clear solution while warm and a solid white paste while at room temperature.

#### Synthesis of Cs_2_Na_1–*x*_Li_*x*_In_0.9_Sb_0.1_Cl_6_ Nanocrystals

Nanocrystals were synthesized using the procedures developed by Locardi et al.^21^ with several modifications. 0.48 mL (0.24 mmol) of 0.5 M Na-oleate and 0.5 M Li-oleate solutions, 0.9 mL (0.225 mmol) of 0.25 M In-oleate solution, 0.1 mL (0.025 mmol) of 0.25 M Sb-oleate solution, 1 mL (0.5 mmol) of 0.5 M Cs-oleate solution, 0.5 mL (1.52 mmol) oleylamine, 4.5 mL of octadecene (ODE) and a stirring bar were inserted into 20 mL glass vial. The mixture was stirred for 5 min at 60–140 °C in an open-air environment. Then, 200 μL (1.72 mmol) of Bz-Cl was swiftly injected while at elevated temperature (60–140 °C). The reaction was stirred for another 5 s and then cooled in a cold-water bath. The solution was then centrifuged, first at 7000 rpm for 10 min at 16 °C, and the precipitate was redispersed in 5 mL of hexane and then centrifuged at 3500 rpm for 5 min at 16 °C. The solution was transferred to a new tube, centrifuged at 7000 rpm for 10 min at 16 °C, and separated from the residue.

#### Synthesis of Cs_2_In_0.9_Sb_0.1_Cl_5_·H_2_O Nanocrystals

Nanocrystals were synthesized using the procedures developed by Locardi et al.^21^ with several modifications. 0.9 mL (0.225 mmol) of 0.25 M In-oleate solution, 0.1 mL (0.025 mmol) of 0.25 M Sb-oleate solution, 1 mL (0.5 mmol) of 0.5 M Cs-oleate solution, 0.5 mL (1.52 mmol) oleylamine, 4.5 mL of octadecene (ODE), and a stirring bar were inserted into a 20 mL glass vial. The mixture was stirred for 5 min at 120 °C in an open-air environment. Then, 200 μL (1.72 mmol) of Bz-Cl was swiftly injected while at the elevated temperature. The reaction was stirred for another 5 s and then cooled in a cold-water bath. The nanocrystals were separated by using centrifugation according to the procedure described above. Additionally, Cs_2_InCl_5_·H_2_O Nanoparticles were synthesized as a reference. No structural differences were observed, as characterized by X-ray diffraction (XRD) and TEM.

### Sample Characterization Procedures

#### Optical Characterization

The optical characterization was performed using an Agilent BioTek Synergy H1 hybrid multimode reader spectrophotometer with samples loaded in polystyrene 96-well plates and an Edinburgh FLS1000 photoluminescence spectrometer with samples loaded in quartz cuvettes. The xenon lamp was a light source in both cases. All of the measurements were performed with the products inside the hexane emulsion solution and with a reference blank well of clean hexane. All the measurements were performed using a wavelength step size of 0.5 or 1 nm and an intensity gain between 50 and 100, depending on the specific product and the solution concentration. The in situ postsynthesis cationic exchange was performed by adding increasing amounts of Li-oleate from 6 to 175 μmol and then performing optical measurements. The excitation wavelength for the photoluminescence ranged between 320 and 360 nm, depending on the Na–Li composition.

#### X-ray Diffraction (XRD)

The nanocrystals’ solution in hexane was centrifuged at 12,000 rpm for 10 min at 16 °C, and the precipitate was drop-cast onto a rectangular microslip glass substrate (76 × 26 mm^2^) or a p-type silicon wafer slice (10 × 10 mm^2^). Measurements were taken using a Rigaku Smart-Lab 9 kW high-resolution X-ray diffractometer equipped with a rotating anode X-ray source and a HyPix-3000 detector. We used a 1.54 Å (Cu Kα) wavelength with a 2xGe(220) monochromator and a 2θ range of 10–90° with a step size of 0.01°. The in situ heating measurements were conducted using a temperature-dependent attachment stage with a carbon dome in a vacuum environment.

#### Inductively Coupled Plasma Mass Spectroscopy (ICP-MS)

The nanocrystal hexane solutions were dried in glass vials by using a vacuum evaporator (Heidolph Hei-VAP). Then the nanocrystals were dissolved in nitric acid upon heating at 100 °C, diluted with water, and measured using a 7800 ICP-MS equipped with an SPS4 Autosampler (Agilent).

#### Transmission Electron Microscopy (TEM)

A drop of dilute nanocrystals’ solution in hexane was cast onto a TEM grid – carbon film on 300 mesh copper and observed in TEM mode using a FEI/Thermo-Fisher Tecnai G^2^ T20 with LaB_6_ electron source operated at an accelerating voltage of 200 keV.

#### High-Angle Annular Dark-Field Scanning Transmission Electron Microscopy (HAADF-STEM)

A TEM grid was prepared in the same way as for the TEM characterization and observed in HAADF-STEM mode using an FEI/Thermo-Fisher double-corrected 60–300 Titan Themis FEG-S/TEM operated at an accelerating voltage of 200 kV. Energy-dispersive X-ray spectroscopy (EDS) measurements were performed using a Dual-X detector (Bruker).

#### Scanning Electron Microscopy (SEM)

The nanocrystals’ solution in hexane was centrifuged at 12,000 rpm for 10 min, and the precipitate was drop-cast onto a p-type silicon wafer slice (10 × 10 mm^2^). SEM micrographs were taken using an HR-SEM microscope model Zeiss Ultra-Plus. Samples were placed at a working distance of 3.5–4 mm and measured using an acceleration voltage of 1.5–3 kV.

### Density Functional Theory (DFT) Calculations

Ab-initio DFT calculations were performed on Cs_2_Na_1–*x*_Li_*x*_InCl_6_ bulk and surface geometries using the Vienna Ab-Initio Simulation Package (VASP) code.^46^ The Atomistic Simulation Environment (ASE) package^47^ has been used for preprocessing and postprocessing of calculation results. All structural relaxations have been performed within the Generalized Gradient Approximation (GGA) framework using the PBEsol^48^ exchange-correlation functional. Relativistic effects are introduced in our calculations by including spin–orbit coupling (SOC) corrections. The Projector Augmented Waves (PAW)^49^ pseudopotentials are used with a cutoff energy of 520 eV and a Γ-centered *k*-spacing of 0.25 Å^–1^. In the case of the surface calculations, only one *k*-point is sampled in the *z*-direction (the direction in which the vacuum of 18 Å is introduced). We model the (100) surface termination in the cubic and trigonal phases, according to the morphology of the nanocrystals (Figure 1c). Keeping the stoichiometry of the surface for the cubic and the trigonal phases at the nominal value, this results in a CsCl and Na_*x*_Li_1–*x*_InCl_2_ termination for the cubic phase and a CsCl_3_, CsInCl_3_ termination for the trigonal phase. Because of the asymmetry in the surface terminations, we use dipole corrections all throughout our surface calculations.

We used the conjugate gradient algorithm as implemented in VASP, allowing all lattice parameters and ionic positions to relax in the bulk models. For the surface models, however, the lattice is kept fixed as well as the positions of the ions in the middle layers of the slab. Convergence is assumed when the residual forces on all atoms are below 0.05 eV/Å. All VASP input and output files associated with this project are stored in a repository and freely accessible on DTU Data, ensuring reproducibility and reusability of the results.^50^

Formation energies for the bulk are calculated through the energy difference from the sum of the elemental reference state: *E*_form_ = (*E*_bulk,DFT_ – Σ*_i_E*_*i*,DFT_)/*N*, where *E*_bulk,DFT_ is the total energy of the bulk unit cell calculated through the DFT, *E*_*i*,DFT_ is the DFT energy of component *i*, and *N* is the number of atoms in the bulk unit cell. For surfaces, the formation energy is calculated through *γ̅* = (*E*_slab,DFT_ – *N*_slab_·*E*_form_)/2*A*, where *E*_slab,DFT_ is the total energy of the surface slab calculated through the DFT, *N*_slab_ is the number of atoms present in the slab model, *E*_form_ is the total energy of the bulk unit cell calculated through the DFT, given in units of [eV/atom], and *A* is the surface area in [Å^2^], calculated by multiplying the *a* and *b* lattice parameters in the slab model.

## Results

To synthesize the Cs_2_(Li,Na)­In_0.9_Sb_0.1_Cl_6_ nanocrystals, we used the hot injection method, as described in our previous work,^24^ yielding nanocrystals with sizes of tens of nanometers (Figure 1d). The HAADF-STEM and its fast Fourier transform (FFT) show a cubic symmetry of the nanocrystals’ lattice (Figure 1e). Surprisingly, d-spaces of the Cs_2_LiInCl_6_ nanocrystals are larger than those of the Cs_2_NaInCl_6_ nanocrystals,^24^ manifesting a lattice expansion indicating the importance of surface energy.^51–53^

The stable phase of Cs_2_LiInCl_6_ at normal conditions (pressure 1 atm., temperature 293 K) has the trigonal structure, while in the presence of moisture, the Cs_2_InCl_5_·H_2_O hydrate phase forms readily.^43^ We hypothesize that the cubic phase gains stability due to the lower surface energy of the ligand-capped faces of the nanocrystals with a cubic structure compared to other structures, as was shown earlier for CdS and CdSe.^51,54^ Indeed, due to the lattice termination, the surface of a crystal has a different formation energy, which can cause a different structural tendency, especially affecting nanocrystals due to their high surface-to-volume ratio, and resulting in a different stable phase.^55,56^

We used the Cs_2_(Na,Li)­InCl_6_ system to evaluate the mechanisms of phase stabilization through computational methods. Using Density Functional Theory (DFT), we compute the total formation energies for the bulk of cubic and trigonal phases at varying Na–Li alloying ratios in Cs_2_Na_1–*x*_Li_*x*_InCl_6_ (Figure 2a), and the {100}-facet for the same phases (Figure 2b). For the trigonal bulk phase, we model the (001) surface. The surfaces were chosen based on our experimental observations. Cs_2_Na_1–*x*_Li_*x*_InCl_6_ DP nanocrystals form cubic or semicubic shapes with {100}-facets. For the trigonal phase, the bulk sample’s SEM micrograph shows faceted microcrystals with specific polyhedron morphologies, including mostly flat rhombus microcrystals (Figure 1c) or decahedrons with trapezoid and pentagonal exposed facets. Therefore, the morphology and shape of the microcrystals can indicate the trigonal phase, growing according to the trigonal (001) plane.

**Figure 2:**
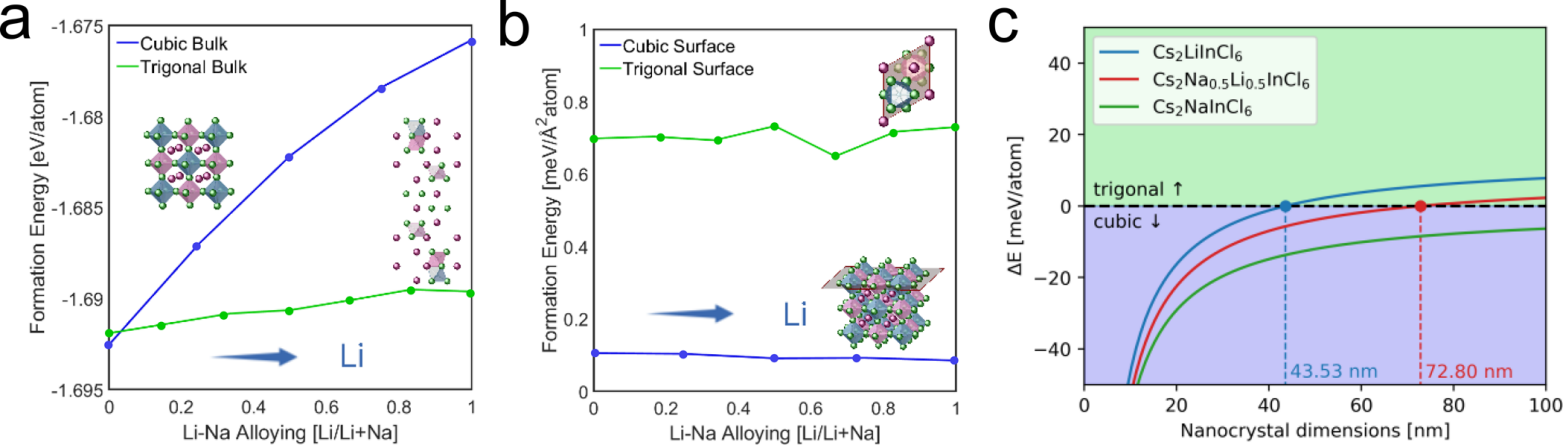
Formation energy of the cubic and the trigonal phases of Cs_2_Na_1–*x*_Li_*x*_InCl_6_ for (a) bulk and (b) {100} surface. Insets show the corresponding structures. (c) Formation energy difference between the cubic and trigonal phases, as a function of the NC size. Negative energy difference indicates the stability of the cubic phase. Zero energy difference indicates a phase transition (as shown for Cs_2_LiInCl_6_ and Cs_2_Na_0.5_Li_0.5_InCl_6_).

The formation energy of the bulk cubic phase increases faster with Li content than the formation energy of the trigonal phase (Figure 2a), showing that a pure Cs_2_NaInCl_6_ composition will adopt the cubic structure in bulk form, but the trigonal phase is preferred almost instantly when Li is introduced to the system (the details of the methodology are reported in the SI). This observation is in agreement with experimental findings for bulk Cs_2_NaInCl_6_ and Cs_2_LiInCl_6_ compositions.^22,56^ The formation energies of the {100} surfaces of these phases, on the contrary, show no prominent dependence on the Na–Li alloying ratio, and the cubic surface energy is lower than that of the trigonal for all alloying compositions (Figure 2b). We can assess the relative stability of the two phases across different NC sizes by considering surface and bulk contributions (Figure 2c), as described in SI. At small NC sizes, the cubic phase dominates because its surface formation energy is more favorable than that of the trigonal phase. However, as the NC size increases, the bulk contribution becomes more significant, and the lower bulk formation energy of the trigonal phase drives the phase transition. For Cs_2_LiInCl_6_, we find this transition point at an NC size of 43.53 nm, and for Cs_2_Na_0.5_Li_0.5_InCl_6_ at 72.80 nm. For CsNaInCl_6_, the cubic phase is always preferred.

We synthesized nanocrystals with varying Na–Li alloying ratios to experimentally verify the stabilization of the cubic phase in the nanocrystals. To ensure the presence of both Li and Na in the nanocrystals, we performed STEM-EDS measurements (Figure S2a–f). While EDS cannot detect Li directly, its presence can be claimed from the concentration of other elements and the assumption of charge neutrality of the analyzed compounds (Table S1). We then performed inductively coupled plasma mass spectroscopy (ICP-MS) to validate Li^+^ insertion into the lattice (Figure S2g). The measured Li concentration increases with increasing Li precursor content in the reaction, while the Na signal intensity decreases, indicating Li incorporation in the sample at the expense of Na. The indirect Li assessment by STEM-EDS, together with ICP-MS analysis, supports Li incorporation within the NCs and successful alloying between Li and Na.

Atomic-resolution STEM reveals nanocrystals with a cubic lattice for Na–Li compositions from purely Na-based to purely Li-based (Figure 3a–c, NCs are oriented in the [100] zone axis), which rules out the triclinic phase, stable in the bulk. The XRD data for nanocrystals with varying Na–Li alloying ratios support the above-mentioned conclusions. The peaks of the 100% Li XRD pattern (Figure 3d, top) correspond to the cubic Cs_2_LiInCl_6_ phase with an expanded lattice parameter compared to the theoretical one (mp-1113017).^58,59^ The XRD diffraction peaks shift to lower Q-values (larger *d*-spacing) with increasing Li concentration (Figure S11). All compositions exhibit lattice parameters exceeding that of the calculated phase (10.51 Å); this agrees with TEM data, also showing an increased lattice parameter of 11.08 Å (Figure 1d). Additionally, we use a CIF reference, which follows the same double perovskite structure pattern, adjusted to the experimentally observed lattice parameter of 11.08 Å (Figures 3d, bottom; S6b). The high degree of agreement between this modified CIF and the experimental data clearly indicates that the double perovskite structure was obtained.

**Figure 3:**
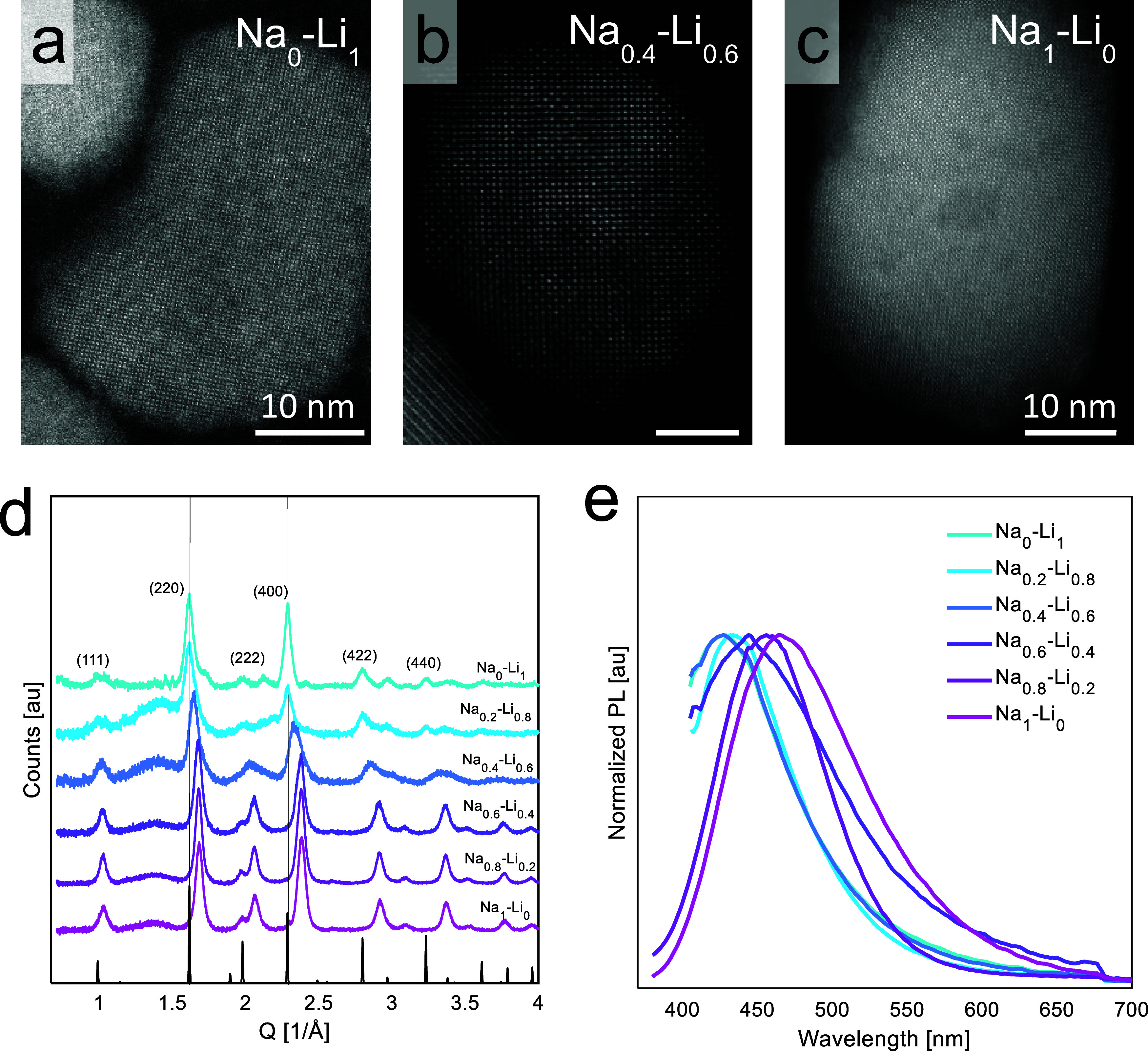
(a–c) STEM images of the NCs with different Na–Li alloying. (d) XRD of Cs_2_Na_1–*x*_Li_*x*_In_0.9_Sb_0.1_Cl_6_ with the theoretical diffraction peaks of the cubic phase (mp-1113017) modified to the 11.08 Å expanded lattice parameter, guiding lines added to the two most intense ones. (e) Normalized PL of Cs_2_Na_1–*x*_Li_*x*_In_0.9_Sb_0.1_Cl_6_. Na_1_Li_0_ and Na_0.8_Li_0.2_ under the excitation of 320 nm; Na_0.6_Li_0.4_, Na_0.4_Li_0.6_, Na_0.2_Li_0.8_, and Na_0_Li_1_ under the excitation of 360 nm.

The samples with a higher Li content demonstrate contamination indicated by smaller-intensity diffraction peaks near the diffraction peaks derived from the main cubic phase, which could be assigned to the Cs_2_InCl_5_·H_2_O phase (Figure S6a,b). The hydrate phase was inevitably present, even for reactions performed under inert conditions, and its presence increased with higher Li concentrations. Moreover, we extract the microstrain from all alloyed compositions (Table S2). As expected, the most strained samples are the 0.4Li and 0.6Li because of significantly different radii in the lattice (0.76 Å for Li^+^ and 1.02 Å for Na^+^ for 6-coordinated species).^60^ Samples with higher Li concentrations exhibit a slightly higher strain compared to samples with lower Li concentrations (Table S2). The relatively high microstrain observed across all compositions may contribute to the unexpected lattice parameter expansion, particularly in the high-Li-content sample, which exhibits lower symmetry in the bulk phase. However, further investigation is required to fully elucidate this relationship.^60^

Photoluminescence emission spectra were measured for all of the compositions. The excitation wavelength was varied from 320 to 360 nm for samples with higher Li content to account for the observed PLE shift (Figure S8). PL emission spectra show a blue shift trend with higher Li concentrations up to 40% Li, reaching the emission band maximum of 445 nm (Figure 3e). The XRD data clearly show the presence of the Cs_2_InCl_5_·H_2_O hydrate phase at higher Li concentrations (above 50%). Furthermore, we synthesized the pure hydrate phase to confirm its optical properties (Figure S7). The Cs_2_InCl_5_·H_2_O NCs exhibit one emission peak when excited at 320 nm and an additional emission peak at 425 nm when excited at 365 nm.^57^

Although all structural analyses confirm the double perovskite structure, we hypothesize that PL emission from the hydrate phase is stronger overall and thus masks the DP phase contribution. Consequently, the unavoidable hydrate phase prevents direct comparison of XRD and PL emission trends in high-Li-concentration samples.

From the XRD peak analysis, we confirm the NC’s size as tens of nanometers (Table S2), matching the TEM micrograph results (Figure S3). The nanocrystals’ sizes vary for different alloying ratios, but there is no clear trend. Thus, the optical blue shift trend is related to the Na–Li ratio rather than the NC size.

HAADF-STEM micrographs of three compositions with increasing Li content (0, 60, and 100%) in the [100] zone axis were used to calculate strain distribution using geometric phase analysis (GPA)^61^ in Strain++ software^62^ (Figure 4). The strain is extracted at each point compared to the ideal planes of the analyzed crystalline structure; strain in the *x* direction ε_*xx*_, strain in the *y* direction ε_*yy*_, and shear strain ε_*xy*_ are calculated. The strain trend from the GPA analysis is in agreement with the XRD peak analysis (see the SI). The lattice nonhomogeneous strain is the highest in the most strongly alloyed composition (the NC, containing both Na and Li), which we attribute to unit-cell fluctuations arising from higher-order alloying.^60^

**Figure 4:**
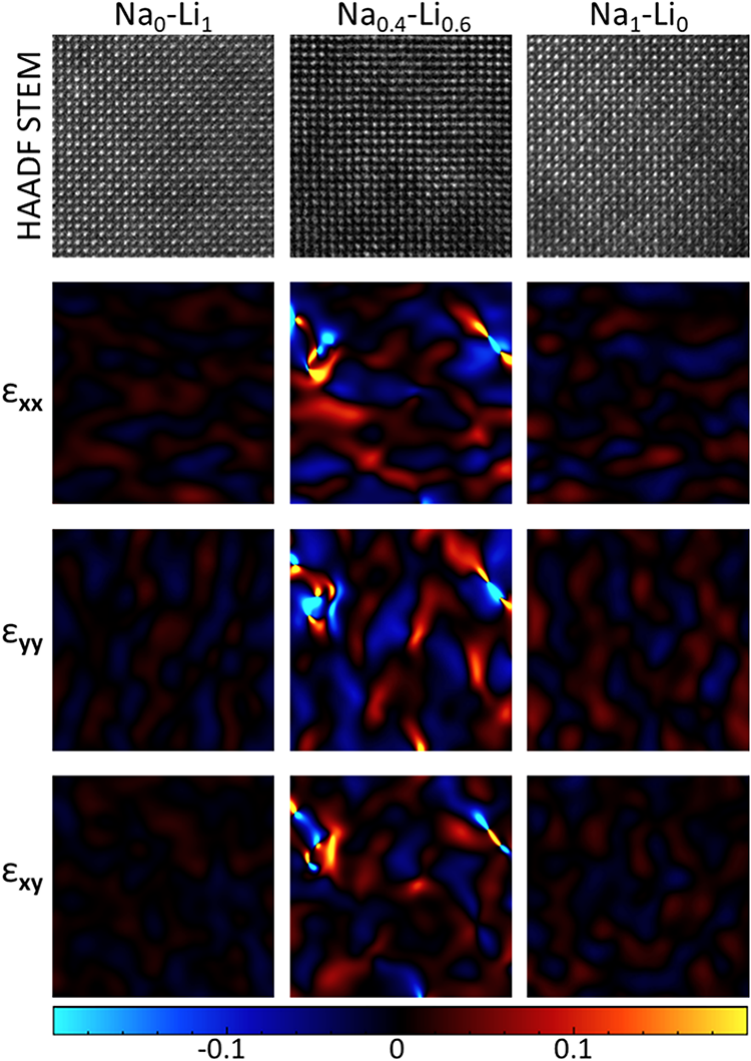
HAADF-STEM micrographs of 10 × 10 nm^2^ central regions of Cs_2_Na_1–*x*_Li_*x*_In_0.9_Sb_0.1_Cl_6_ NCs oriented in the [100] zone axis (*x* = 0, 0.6, and 1), and distributions of strain along different directions (ε_*xx*_, ε_*yy*_, and ε_*xy*_), calculated using GPA.

We finally tested an alternative approach to fabricating alloyed samples through ion exchange. This approach is well-known for halide exchange in lead halide perovskites.^62–64^ The soft lattice and small size of halide perovskite NCs, and probably the small size of Li^+^, enable fast cation exchange. We added Li^+^ precursor to Cs_2_NaIn_0.9_Sb_0.1_Cl_6_ nanocrystals; as a result, the PL peak blue-shifted (Figure 5a). The dependence of the emission band position on the Na–Li ratio allows the use of the nanocrystals for sensing Li^+^ ions in the surroundings. PL dependence on the added amount of Li^+^ precursor demonstrates sensitivity in the range of 2 orders of magnitude of added Li, from 6 to 175 μmol (Figure 5b). The range could be further increased by using different concentrations of Cs_2_NaIn_0.9_Sb_0.1_Cl_6_ nanocrystals. We hypothesize that the sensitivity range can also vary with surface modifications and the surrounding solvent.

**Figure 5:**
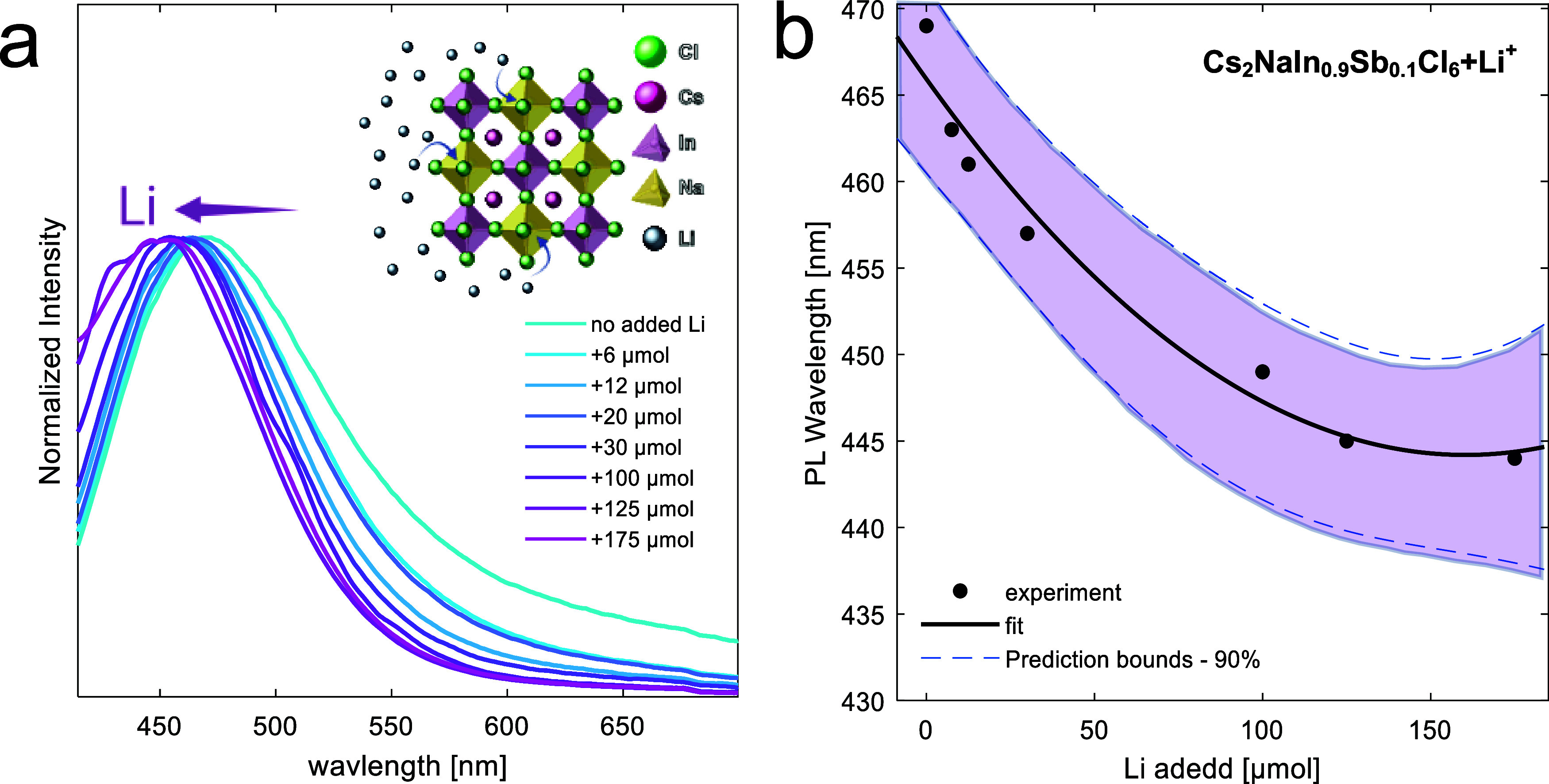
(a) Normalized PL of Cs_2_NaIn_0.9_Sb_0.1_Cl_6_ NCs, to which gradually increasing volumes of Li-containing solutions were added. Inset: Cs_2_NaIn_0.9_Sb_0.1_Cl_6_ structure in Li^+^ ions surrounding, demonstrating Na-to-Li postsynthetic cationic exchange. (b) Calibration curve of the PL maximum wavelength with the addition of Li.

## Discussion

To suppress the formation of the orthorhombic Cs_2_InCl_5_·H_2_O hydrate, it is critical to perform the synthesis using rigorously dried solvents and low-humidity conditions. However, higher Li concentrations inherently promote this competing structure, which we could not completely eliminate. For bulk Cs_2_LiInCl_6_, it was shown that the competing hydrate phase is easily formed.^43^ We note in passing that other Cs–In containing compositions also suffer from this competing phase, indicating its general thermodynamic stability.^23^ It thus emphasizes the importance of enhanced stability of the cubic phase, which is demonstrated in nanocrystals. Optical properties of this hydrate phase with perovskite-derived structure were reported previously, both in bulk and nanocrystalline form, particularly when doped with Sb.^63–65^ We have observed the increased formation of hydrate nanocrystals when syntheses were conducted at ambient conditions in up to 80% humidity or using long-stored solvents without prior drying (even when synthesis is under inert conditions) (Figures S4 and S5). Therefore, careful validation was necessary to confirm the presence of Li-based double perovskite nanoparticles.

Atomic-resolution HAADF-STEM micrographs and their corresponding FFT showed a well-defined cubic symmetry, consistent with the expected structure of a cubic perovskite (Figures 1 and 2). This local method is supported by three integral methods: (i) XRD demonstrates an identical set of main diffraction peaks for the whole Cs_2_(Li,Na)­In_0.9_Sb_0.1_Cl_6_ series, matching the cubic phase with continuously changing lattice parameters. (ii) Compositional analysis using ICP-MS confirmed the presence of Li in the samples. This is further supported by STEM-EDS measurements, which indirectly indicate the presence of Li, which the hydrate phase does not contain. (iii) Finally, adding Li precursor to Na-based perovskite caused a blue shift in the emission, which was reported for Li-containing halide perovskites.^66^ As previously shown, differences in ionic radii and microstrains (octahedral tilting or rotation) both directly influence the PL emission shift trend.^24^ Meanwhile, the characteristic hydrate emission was observed for the samples, and the hydrate phase was identified by XRD (Figure S6). At higher Li concentrations (60% and above), we hypothesize that the hydrate phase emission is more dominant, and overall, the Li-containing DP emission is less pronounced.

Notably, even for samples consisting of a dominating hydrate phase, one can still find small NCs with cubic symmetry in the TEM micrographs (SI, Figure S5). That suggests that the small size of nanocrystals stabilizes the DP cubic form of Cs_2_LiInCl_6_ and prevents the formation of Cs_2_InCl_5_·H_2_O hydrate. Similar stabilization against decomposition provided by the high surface-to-volume ratio of nanocrystals was recently demonstrated in the case of Cs_2_AgSbBr_6_ and Cs_2_AgSbI_6_ by Horani and Gamelin.^67^

## Conclusions

To conclude, we have synthesized Cs_2_LiIn_0.9_Sb_0.1_Cl_6_ and Cs_2_(Li,Na)­In_0.9_Sb_0.1_Cl_6_ nanocrystals. The resulting phase exhibits cubic symmetry according to HRTEM, with XRD patterns consistent with the double perovskite structure. This is in agreement with DFT predictions of the stabilization of the cubic phase in smaller particles and the trigonal phase in a bulk form. PL emission blue shifts with the increase of Li content up to 40% Li, which differentiates the Li-containing perovskite phase from the easily formed hydrate. For higher Li concentration, the hydrate emission is more dominant.

Nanocrystal of the Cs_2_Na_0.4_Li_0.6_InCl_6_ composition demonstrates a higher strain in the nanocrystal’s volume, ascribed to the large size difference of Li^+^ and Na^+^. Nevertheless, the stability of the cubic phase over the whole range of compositions allows Li doping through cation exchange. We exemplified how this can provide the possibility to sense Li^+^ cations in the surrounding environment.

This work features Li-based double perovskite nanocrystals, providing insights into the formation and identification of competing phases. It also brings about the vision of in situ monitoring of Li ions for battery technology.

## Supplementary Material



## Notes

The descriptions of GPA workflow, STEM-EDS analysis, size distribution analysis, secondary hydrate phase characterization, XRD peak analysis, details of DFT calculations, and supporting results (PDF)

## #

University of Toronto, 27 King’s College Circle, Toronto, Ontario M5S 1A1, Canada (sabbatical)
