# Supplementary material for: Surface Stabilization of the Cubic Phase in Lithium Alloyed Double Perovskite Nanocrystals
Source: Chem Mater. 2026 Mar 31;38(7):3218–26. doi: 10.1021/acs.chemmater.5c02730 (PMC13084993; doi:10.1021/acs.chemmater.5c02730)
Supplement: Supplementary file 1 [file cm5c02730_si_001.pdf]

Supporting information

Surface Stabilization of the Cubic Phase in Lithium Alloyed Double Perovskite Nanocrystals

Saar Shaek [a,†], Offir Zachs-Maishlos [a,†], Lotte Kortstee [d], Rachel Lifer [a], Emma H. Massasa [a], Georgy Dosovitskiy [a,b], Yaron Kauffmann [a], Boaz Pokroy [a], Ivano E. Castelli [d], Yehonadav Bekenstein [a,b,c,*]

[a] Department of Materials Science and Engineering, Technion – Israel Institute of Technology, 32000 Haifa, Israel.

[b] Solid State Institute, Technion – Israel Institute of Technology, 32000 Haifa, Israel.

[c] The Resnick Sustainability Center for Catalysis Institution, Technion – Israel Institute of Technology, 32000 Haifa, Israel.

[d] Department of Energy Conversion and Storage (DTU Energy), Technical University of Denmark, Agnes Nielsens Vej 301, DK-2800 Kongens Lyngby, Denmark.

[†] Equal contribution

Supplementary methods

Geometric phase analysis (GPA):

We calculated the strain within the nanoparticles using geometrical phase analysis (GPA) for high-resolution S/TEM micrographs [1] with Strain++ software.[2] The analysis is based on comparing the phases of the components of the real image extracted from the fast Fourier transform (FFT) to a set of ideal unstrained lattice planes. This way, the technique quantifies local changes in the periodicity of crystalline materials, making it robust against noise and contrast variations and effective for precise strain analysis. All HRSTEM micrographs were acquired under very strict conditions to avoid any sample drift and beam damage during scanning – low dose combined with very fast acquisition and in some cases multiple frames averaging.

The procedure for the method is following: We load an HR-S/TEM micrograph (we used micrographs of nanocrystals oriented in the [100] zone axis) and then transform it into frequency space using a Fourier transform. We select specific crystallographic reflections (g-vectors) from the Fourier-transformed micrograph, and the software applies masks to isolate the features of interest. Then, for each point of the image, the phase information for these features is compared to the ideal g-vectors, corresponding to a set of ideal planes after an inverse transform. This phase data is then differentiated to generate strain maps and reveal variations in atomic spacing. We used the software visualization tools to interpret the results and to export the strain maps. The workflow is shown in Figure S1.

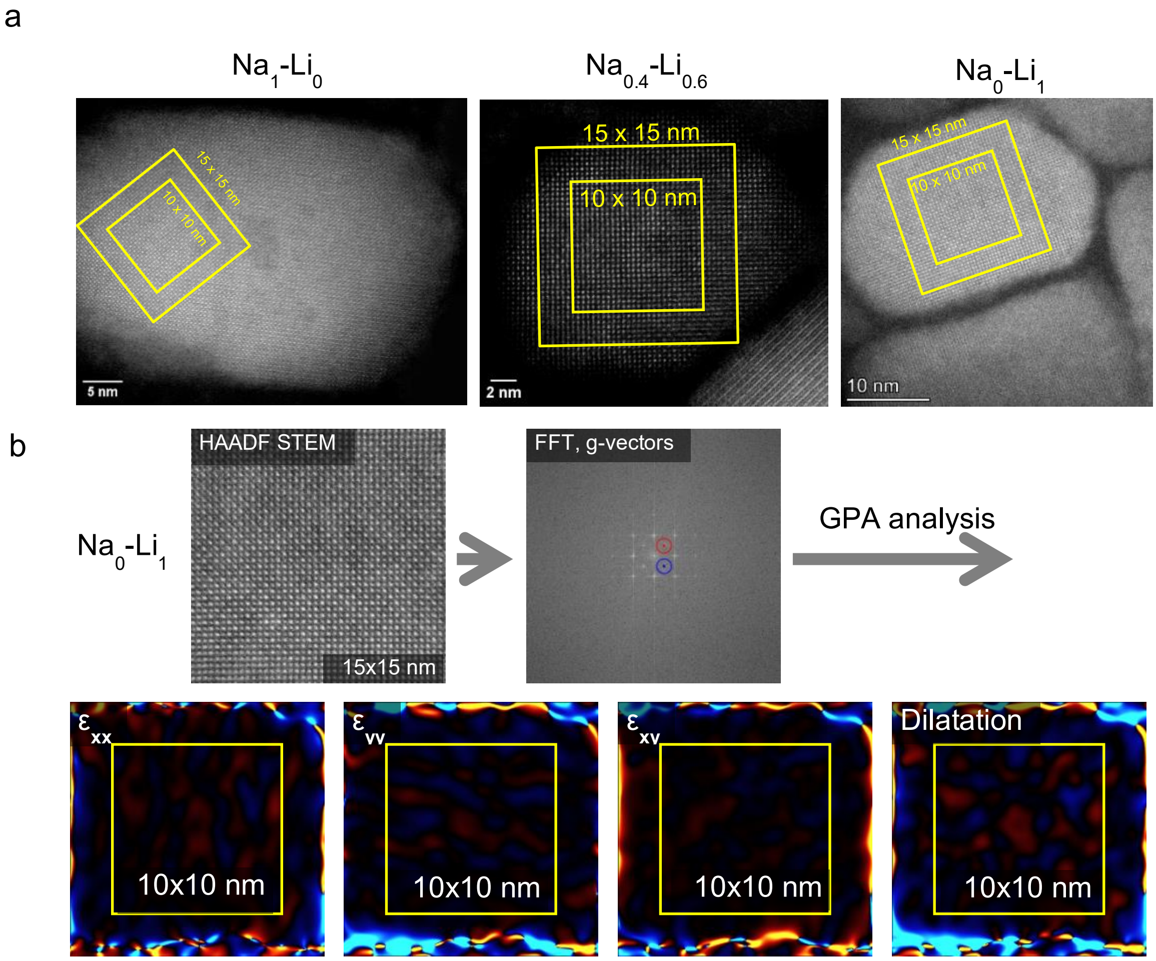

Figure S1. (a) HAADF-STEM micrographs of Sb-doped $Cs_2Na_{1-x}Li_xIn_{0.9}Sb_{0.1}Cl_6$ NCs, where x=0, 0.6, and 1, with marked regions taken for the GPA analysis. (b) The sequence of operations for the GPA analysis. First 15 x 15 nm square regions were cut from HAADF STEM images and stretched or compressed to match the size of 512 pixels. Then GPA analysis was performed using Strain++ software according to the procedures specified by the developer. Central areas of the images were used for g-vectors refinement. Then, 10 x 10 nm regions were cut from the images of strain distributions to remove edge effects.

Calculation of phase transition point in nanocrystals:

To calculate the size of a nanocrystal d_{trans} where a hypothetical transition from cubic structure to trigonal structure takes place, we determine the total energy of nanocrystals as a function of their size for both the cubic and trigonal phases. The transition point is found at a crystal size d_{trans}, where the difference of the total energy of the nanocrystal in the cubic and trigonal phases is 0. In our model, we assume cubic-shaped nanoparticles with (001) surfaces and with edge dimension d. The total energy is a function of both the bulk and surface energetic contributions.

Since the cubic and trigonal phases have different atom packing densities, it is important to express the total energy in units of eV/atom, to ensure we are comparing an equal number of atoms in the bulk of the nanocrystal. The total energy of a cubic NC is described in Eq. S1a, whereas the total energy of a trigonal NC is described in Eq. S1b.

$$E_{total}^{cubic}\left[\frac{eV}{atom}\right] = E_{form}^{bulk,cubic}\left[\frac{eV}{atom}\right] + \frac{6 \cdot E_{form}^{surf,cubic}\left[\frac{eV}{Å^2}\right] \cdot d^2[Å^2]}{\frac{N}{V}\left[\frac{atoms}{Å^3}\right] \cdot d^3[Å^3]} \qquad \text{Eq. (S1a)}$$

$$E_{total}^{trigonal}\left[\frac{eV}{atom}\right] = E_{form}^{bulk,trigonal}\left[\frac{eV}{atom}\right] + \frac{6 \cdot E_{form}^{surf,trigonal}\left[\frac{eV}{Å^2}\right] \cdot d^2[Å^2]}{\frac{N}{V}\left[\frac{atoms}{Å^3}\right] \cdot d^3[Å^3]} \qquad \text{Eq. (S1b)}$$

where E_{form}^{bulk} is the formation energy of the cubic or trigonal bulk in [eV/atom], E_{form}^{surf} is the formation energy of the cubic or trigonal surface in [eV/Å^2], and N and V are the number of atoms and volume [Å^3] of a bulk unit cell in the bulk and trigonal phases.

We calculate the energies of $E_{total}^{cubic}\left[\frac{eV}{atom}\right]$ and $E_{total}^{trigonal}\left[\frac{eV}{atom}\right]$ for three compositions: $Cs_2LiInCl_6$, $Cs_2Na_{0.5}Li_{0.5}InCl_6$ and $Cs_2NaInCl_6$. For each NC dimension *d*, we calculate the difference in energy between the two phases $\left(E_{total}^{cubic}\left[\frac{eV}{atom}\right] - E_{total}^{trigonal}\left[\frac{eV}{atom}\right]\right)$ and plot this energy difference as a function of the nanocrystal size. We find that the energy difference is 0 (i.e., the phase transition occurs) at 43.53 nm for $Cs_2LiInCl_6$ and 72.80 nm for $Cs_2Na_{0.5}Li_{0.5}InCl_6$. With increasing *d*, the energy difference asymptotically approaches the difference of the formation energies for bulk phases. Therefore, we don't find a phase transition point for the $Cs_2NaInCl_6$ composition, as we predict that both the bulk and surface energy in the cubic phase are always predicted to be lower than in the trigonal phase (Figures 2a and 2b). Worth noting that the estimation above addresses the relative stability of the two phases and does not tell anything about the stability of a nanocrystal at all. Whereas below a threshold size for nucleation (different for each phase), the nanocrystal will not be stable anymore. Thus, at very small nanocrystal sizes, the estimation described above is no longer valid, and the apparent infinite benefit of the cubic phase over the trigonal phase with *d* approaching zero has no physical meaning.

Supplementary results

Chemical analysis

To ensure the presence of both Li and Na in the nanoparticles, we performed STEM-EDS measurements (Figure S2a-f and Table S1). As EDS is unable to detect Li, we compare the elements for charge neutralization. Cl anions, per the cation valency, should accompany every metal cation in the composition; that is why Cl is the best element to perform normalization to estimate the ratio of cations. As presented in Table S1, there is an absence of cations for charge neutrality, which is compatible with the inserted Li content in the sample.

We performed inductively coupled plasma mass spectroscopy (ICP-MS) to validate Li cations' insertion into the lattice. The nanocrystals for analysis were separated by centrifugation and then dissolved in nitric acid. The signals of the analyzed elements were first normalized to the joint content of Cs and In (as these elements' content didn't change over the sample series). Then, end members of the series (100% Li and 100% Na) were used to calculate correction coefficients for every element. Thus, the relative signal for each element was obtained. Normalizing to the sum of these signals allowed us to calculate the fraction of each element in the composition. The Li concentration intensity increased with the Li precursor content in the reaction, while the Na concentration intensity decreased, validating the alloying between Li and Na (Figure S2 g).

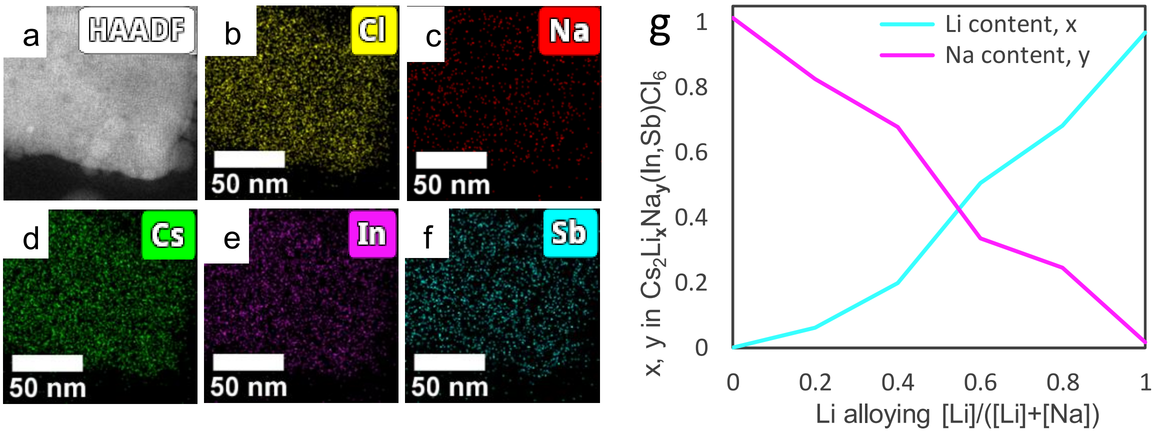

Figure S2. (a) HAADF-STEM and (b-f) STEM-EDS of Sb-doped $Cs_2Na_{0.4}Li_{0.6}In_{0.9}Sb_{0.1}Cl_6$ nanoparticles. (g) Li and Na content measured by ICP-MS in the series of samples of Sb-doped $Cs_2Na_{1-z}Li_zInCl_6$ nanoparticles, where x=0, 0.2, 0.4, 0.6, 0.8, 1. Measured Li and Na content x, y was obtained by normalizing the results to their summary content.

Table S1. STEM-EDS analysis of $Cs_2Na_{0.4}Li_{0.6}In_{0.9}Sb_{0.1}Cl_6$ nanoparticles, where At% and At% σ are the atomic percent results and standard deviation, the At% Pe is the atomic percent excluding the carbon, the At%, norm. [Cl] column is the atomic percentage, normalized to the expected Cl content, and the Reaction Content is the inserted concentration of each precursor element.

	**At%**	**At% σ**	**At% Pe**	**At%, norm. [Cl]**	**Reaction Content**
**C**	63.2	2.80	-		**-**
**Na**	1.38	0.294	3.75	3.54	**0.4**
**Cl**	23.4	3.30	63.63	60.00	**6**
**In**	3.92	0.456	10.65	10.05	**0.9**
**Sb**	0.517	0.0660	1.4	1.33	**0.1**
**Cs**	7.56	0.831	20.56	19.38	**2**
**Li**	-	-	-		**0.6**

Size distribution analysis

We extracted the samples' particle sizes with varying alloying concentrations from the TEM micrographs (Figure S3).

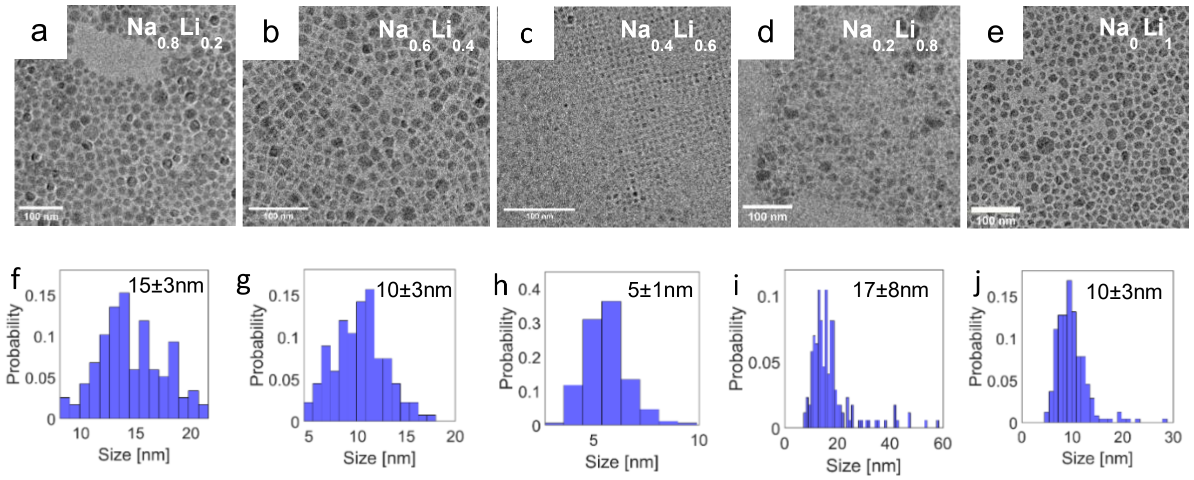

Figure S3. TEM micrographs (a-e) and the additional particle size distribution (f-j) of $Cs_2Na_{1-x}Li_xIn_{0.9}Sb_{0.1}$Cl6 nanoparticles (x=0, 0.2, 0.4, 0.6, 0.8, 1).

Stability and Characterization of $Cs_2InCl_5{\cdot}H_2O$ orthorhombic phase

Our investigation into the synthesis of $Cs_2LiIn_{0.9}Sb_{0.1}Cl_6$ nanocrystals revealed the emergence of a significant competing phase, which we have identified to be isostructural to the orthorhombic $Cs_2InCl_5{\cdot}H_2O$ with structure belonging to the Pnma space group (e.g., PDF 04-009-3832) (Figure S4c). Emission properties of this phase (discussed later) indicate the presence of Sb doping, so we denote its composition as $Cs_2In_{0.9}Sb_{0.1}Cl_5{\cdot}H_2O$, maintaining the same Sb doping as in the perovskites. The appearance of this phase is closely linked to specific environmental conditions and precursor handling. For example:

1. Syntheses conducted under high-humidity conditions (specifically during summer humid months) showed a higher propensity for hydrate formation.
2. The use of solvents that had not undergone rigorous prior drying further promoted the stabilization of the hydrate phase, even when working under otherwise inert conditions.
3. We observed that the presence of Li (relative to Na) fundamentally promotes the formation of this hydrate phase, making its presence appear in high-Li concentration samples.

To assess the relative stability of these two nanocrystals phases, we subjected a mixture of $Cs_2LiIn_{0.9}Sb_{0.1}Cl_6$ and hydrate nanoparticles to thermal treatment at 300°C for 30 minutes

(Figure S4). This process resulted in the following observations. The organic surface ligands decomposed, causing the nanoparticles to fuse into a bulk material. Post-fusion XRD analysis showed that the final product consisted entirely of $Cs_2In_{0.9}Sb_{0.1}Cl_5 \cdot H_2O$. This shows that in bulk form, neither the cubic nor the trigonal symmetry of the $Cs_2LiInCl_6$ composition remains. The hydrate phase's dominance was confirmed by the existence of narrow XRD peaks, demonstrating its high thermodynamic stability in bulk form compared to the lithium-based double perovskite.

This observation further highlights that while the high surface to volume ratio of nanocrystals can stabilize the cubic double perovskite phase, the hydrate remains a persistent and energetically favorable competitor, particularly as particle size increases or environmental moisture is present.

Figure S5 demonstrates additional examples of the nanocrystals, fabricated at temperatures from 60 to 140°C, and resulting in various nanocrystal sizes (Figure S5a-c), and containing mainly the hydrate phase (Figure S5d). However, even within these samples, it was possible to find some small nanocrystals with cubic symmetry. The other explanation may be a phase transformation during the XRD sample preparation, which caused longer exposure of the samples to the ambient conditions than the TEM sample preparation procedure.

Hydrate nanocrystals were purposefully synthesized, their XRD is presented in Figure S6a side by side with the XRD of perovskite nanocrystals containing only hydrate impurities (Figure S6b). Figure S7 demonstrates a typical PL and PLE spectra of a hydrate sample. Different PL emission peak positions are reported in the literature for $Cs_2InCl_5 \cdot H_2O:Sb^{3+}$: Jing et al.[3] report maximum at 580 nm for a bulk phase, Kshirsagar et al.[4] report 2.35 eV (528 nm), and Gong et al.[5] report 596 nm for $Cs_2InCl_5 \cdot H_2O:Sb^{3+}$. As one can see, our results agree with the latter value.

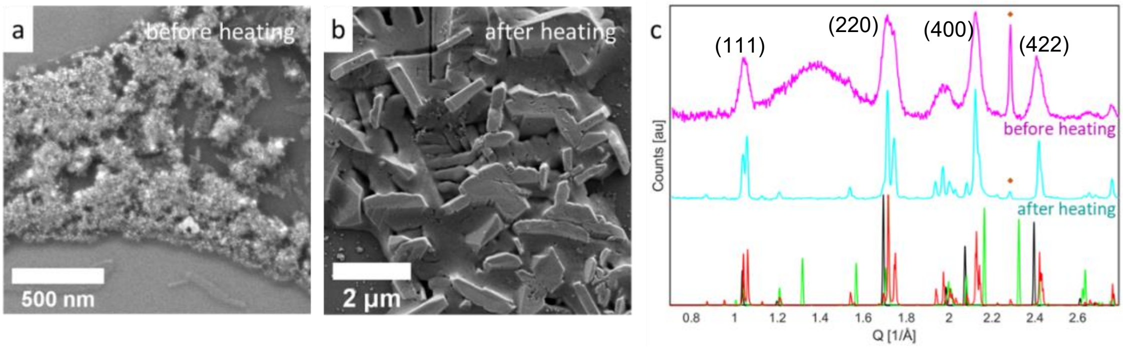

Figure S4. SEM micrograph of (a) mixture of $Cs_2LiIn_{0.9}Sb_{0.1}Cl_6$ and $Cs_2In_{0.9}Sb_{0.1}Cl_5$-H_2O nanoparticles as-synthesized and (b) the sample after heat treatment at 300°C for 30 minutes in situ inside the diffractometer. The micrographs were obtained using secondary electron detection mode. (c) XRD patterns before and after the heating. The theoretical cubic (black, Materials Project mp-1113017), trigonal (green, Materials Project mp-571527), and orthorhombic hydrate phase (red, ICDD PDF #PDF 04-009-3832) peaks are added. The orange diamond marks In-Li-Cl salt. Peak assigning refers to the DP cubic pattern.

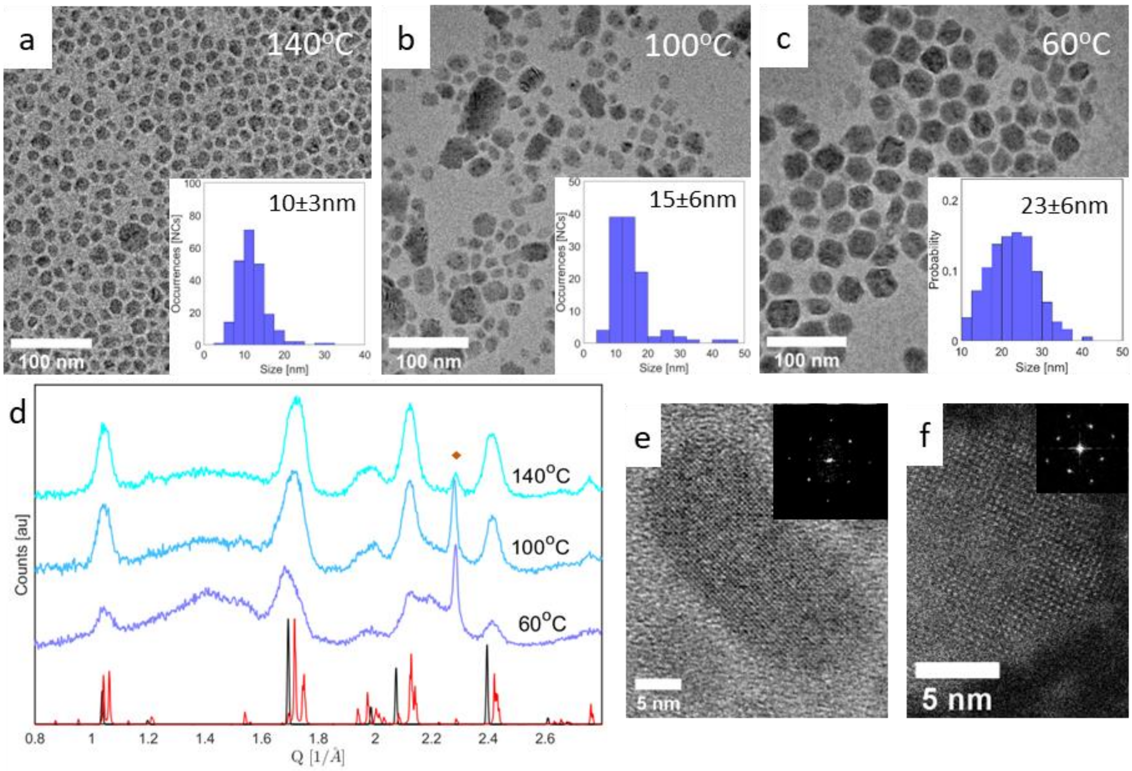

Figure S5. TEM micrographs and size distributions of mixed $Cs_2LiIn_{0.9}Sb_{0.1}Cl_6$ and $Cs_2In_{0.9}Sb_{0.1}Cl_5$-H_2O nanoparticles synthesized at reaction temperatures of (a) 140°C, (b) 100°C, (c) 60°C. (d) XRD of these nanocrystals; the theoretical XRD peaks of the cubic phase (black, Materials Project mp-1113017), orthorhombic hydrate phase (red, ICDD PDF #PDF 04-009-3832), and an orange rhombus marking an In-Li-Cl salt are added. (e), (f) Examples of HRTEM images of the sample synthesized at 140°C, demonstrating nanocrystals with cubic symmetry.

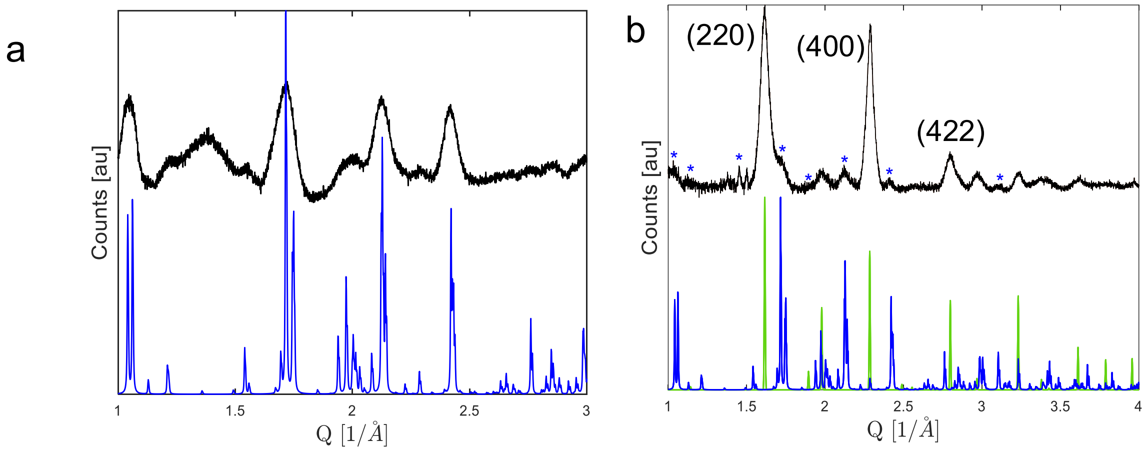

Figure S6. (a) XRD of the $Cs_2In_{0.9}Sb_{0.1}Cl_5$-H_2O hydrate nanocrystal samples (black) and the orthorhombic hydrate phase ICDD PDF #PDF 04-009-3832 (blue). The wide peak between 1 to 1.5 1/Å is an amorphous background resulted of the ligands on the NCs surface. (b) XRD peaks of $Cs_2LiIn_{0.9}Sb_{0.1}Cl_6$ with $Cs_2In_{0.9}Sb_{0.1}Cl_5$-H_2O hydrate secondary phase. The modified $Cs_2LiInCl_6$ CIF (see the main text) in green and the Cs_2InCl_5-H_2O in blue.

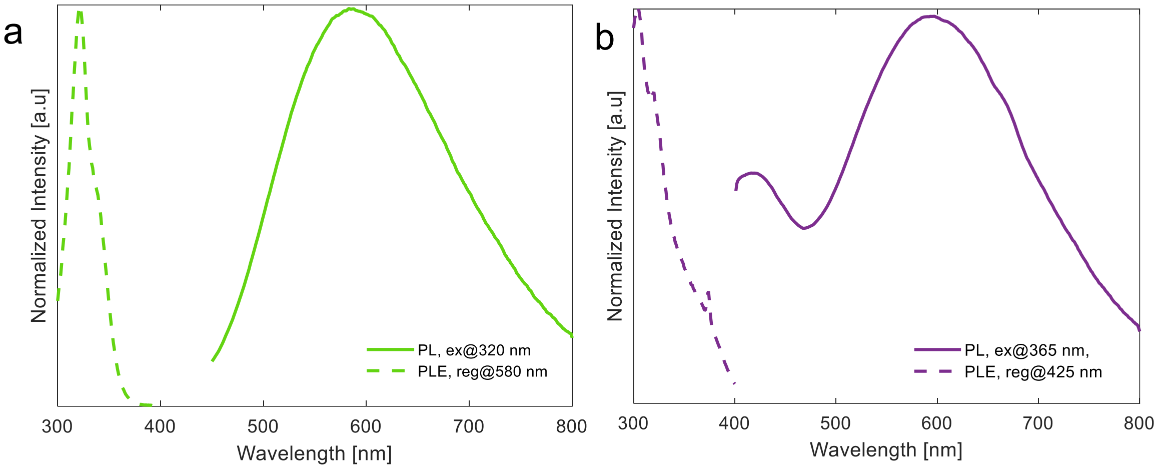

Figure S7. PLE and PL for a pure hydrate phase NCs sample, corresponding to the $Cs_2In_{0.9}Sb_{0.1}Cl_5{\cdot}H_2O$ phase according to the XRD. (a) PL emission excited at 320nm (solid line) and the corresponding PLE (dashed line). (b) PL emission excited at 365nm (solid line) and the corresponding PLE,

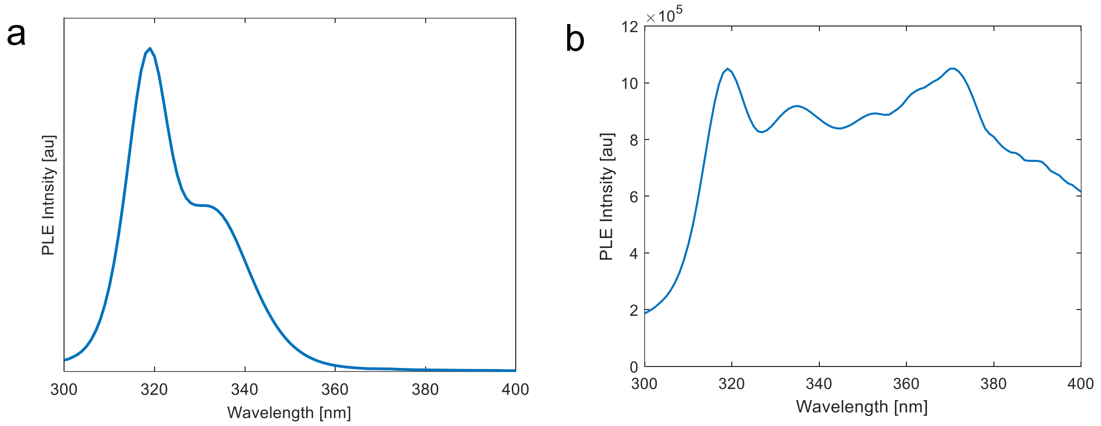

Figure S8. (a) PLE spectrum of $Cs_2NaIn_{0.9}Sb_{0.1}Cl_6$ NCs. (b) PLE spectrum of $Cs_2(Na,Li)In_{0.9}Sb_{0.1}Cl_6$ NCs.

The incorporation of Li into the cubic alloyed composition modified the PLE spectra from the characteristic $^1S_0$–$^3P_1$ transition splitting associated with dynamic Jahn–Teller distortion [6] (Figure S8a) to a more complex PLE profile, featuring intense peaks in the 360–370 nm range (Figure S8b). Moreover, we assume the hydrate phase also contributes to the presented PLE.

Supporting experiments on the ion exchange

As the optical properties of the Na-based double perovskite nanocrystals blueshift with the addition of the Li-oleate precursor, we verify that the changes are not because of the Li-oleate precursor's oleic acid.

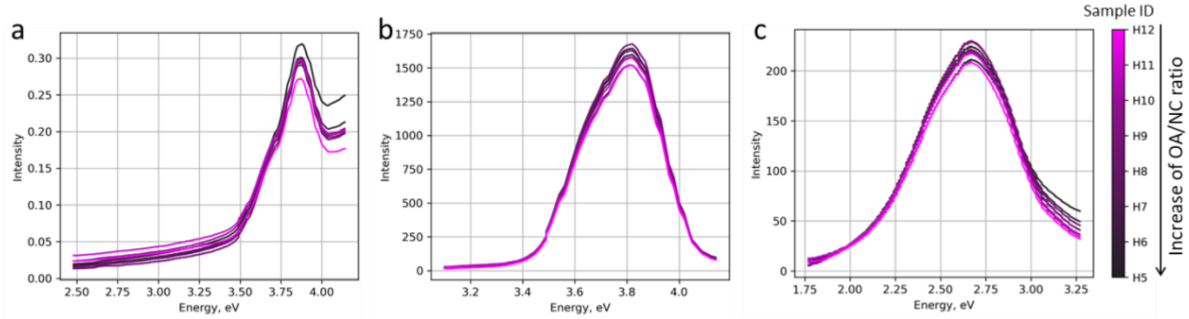

Figure S9. Absorption (a), excitation (b), and emission (c) spectra of $Cs_2NaIn_{0.9}Sb_{0.1}Cl_6$ NCs (50 µl of colloidal solution) with different additions of oleic acid (0.05 to 2 µl for samples H11 – H5, H12 – pure nanocrystals' solution).

Figure 9 shows the absorption, PLE, and PL emission spectra of $Cs_2NaIn_{0.9}Sb_{0.1}Cl_6$ with increasing amounts of oleic acid.

The results indicate that the addition of oleic acid (or M-oleate in the context of the cation-exchange experiment) does not alter the spectral shape or peak positions, except for minor intensity variations. Therefore, the shift of the emission peak observed upon addition of Li-OL is attributed to the incorporation of Li^+ ions.

Alloyed $Cs_2Na_{1-x}Li_xIn_{0.9}Sb_{0.1}Cl_6$- XRD peak analysis

The "fityk" program [7] was used to fit the XRD peaks for the different compositions. The peaks were fitted to a pseudo-Voigt function, and the NCs' size and micro strains were extracted from the Lorentzian and Gaussian contributions, respectively.

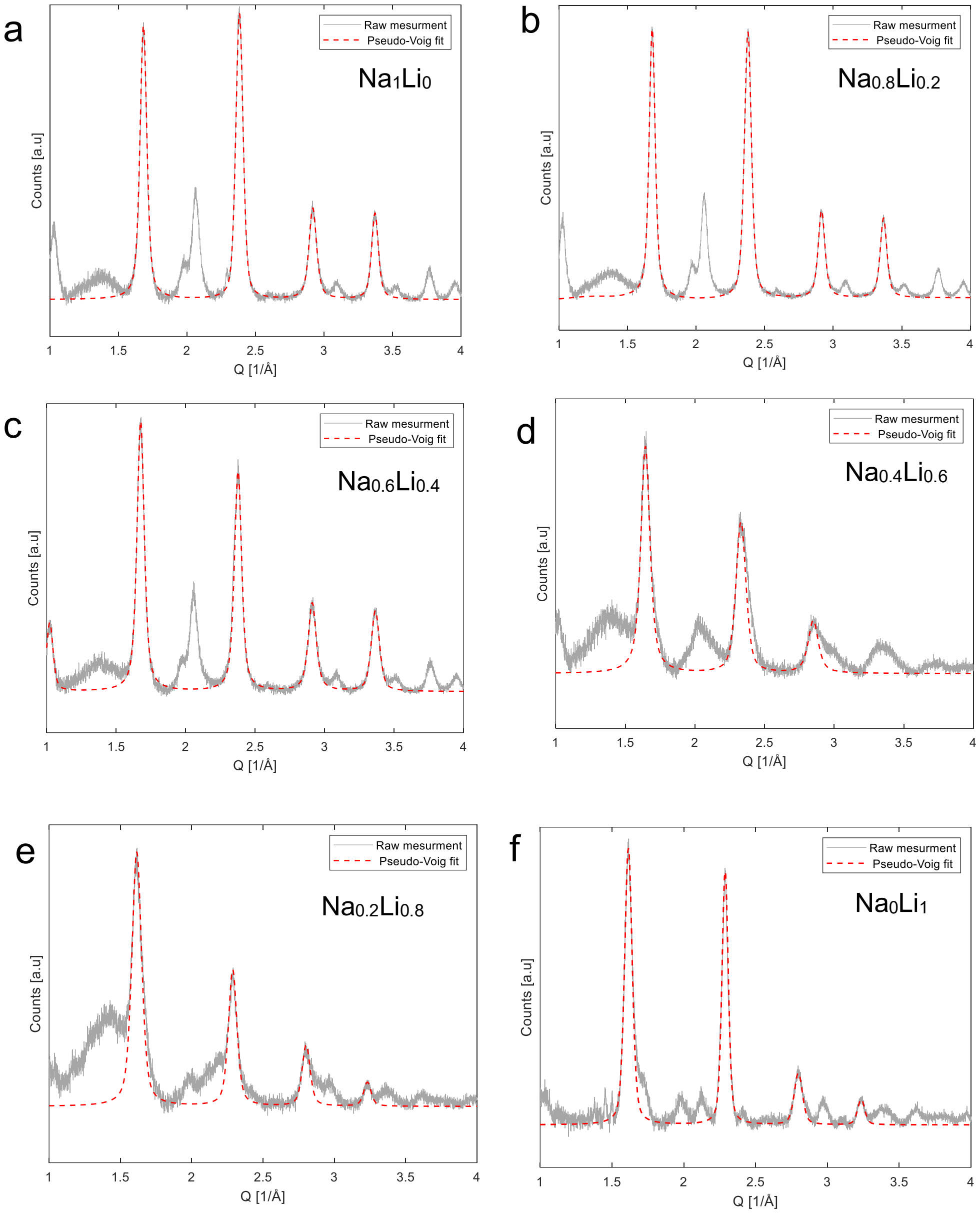

Figure S10. (a-f) XRD peaks of alloyed $Cs_2Na_{1-x}Li_xIn_{0.9}Sb_{0.1}Cl_6$ compositions (in grey) and the peaks that were fitted to a pseudo-Voigt function (red dotted line).

We used the “single line Voight” method to determine the particle size and micro strains. The Scherrer equation was applied to extract the particle size. While the microstrain was obtained from the Gaussian contribution to the peak broadening. Only diffraction peaks that match the double perovskite phase were included in the analysis. Portions of the diffraction profile that exhibited additional broadening due to overlap with adjacent reflections from secondary phases

were excluded from the fitting. Finally, the parameters extracted from the individual peaks were averaged to obtain the mean particle size and microstrain for each composition (Table S2).

Table S2. Pseudo-Voigt peak fit parameters and extracted particle size and micro-strains.

**Sample**	**Average total FWHM**		**Average Gaussian FWHM (W_G)**		**Average Lorentzian FWHM (W_L)**		**Statistics**		**Average particle size [nm]**		**Average microstrain [%]**	
		σ		σ		σ	Red. X^2	Adj.R^2		σ		σ
Na_1Li_0	0.92	0.05	0.66	0.05	0.4295	0.08	60575	0.85	14.4	3	0.78	3E-3
$Na_{0.8}Li_{0.2}$	0.86	0.05	0.600	0.054	0.428	0.037	37243.8	0.84	14	0.9	0.702	1.6E-3
$Na_{0.6}Li_{0.4}$	0.952	0.1	0.6610	0.08	0.479	0.09	17596	0.85	12.7	2.3	1.19	4E-3
$Na_{0.4}Li_{0.6}$	1.226	0.07	0.791	0.1	0.694	0.1	7330.01	0.69	8.7	1.4	1.043	1.9E-3
$Na_{0.2}Li_{0.8}$	1.035	0.08	0.779	0.06	0.437	0.03	10015.1	0.6	13.7	1.4	0.992	4E-3
Na_0Li_1	0.896	0.09	0.7305	0.075	0.2935	0.03	12865.1	0.91	20.4	2.1	0.902	2E-3

Lattice parameter

The lattice parameter was calculated using Bragg's law for all the double perovskite visible peaks for each composition. We use the extracted peak positions after the pseudo-Voigt fit to calculate the average lattice parameter from each composition. Finally, we calculated the cubic unit cell as function of Li concentration (Figure S10).

Table S3. Experimental lattice parameter for each composition.

**Sample**	**Av. lattice parameter [Å]**	
**Na_0Li_1**	**10.55**	0.007
**$Na_{0.8}Li_{0.2}$**	**10.56**	0.009
**$Na_{0.6}Li_{0.4}$**	**10.59**	0.03
**$Na_{0.4}Li_{0.6}$**	**10.79**	0.009
**$Na_{0.2}Li_{0.8}$**	**10.99**	0.008
**Na_0Li_1**	**10.99**	0.009

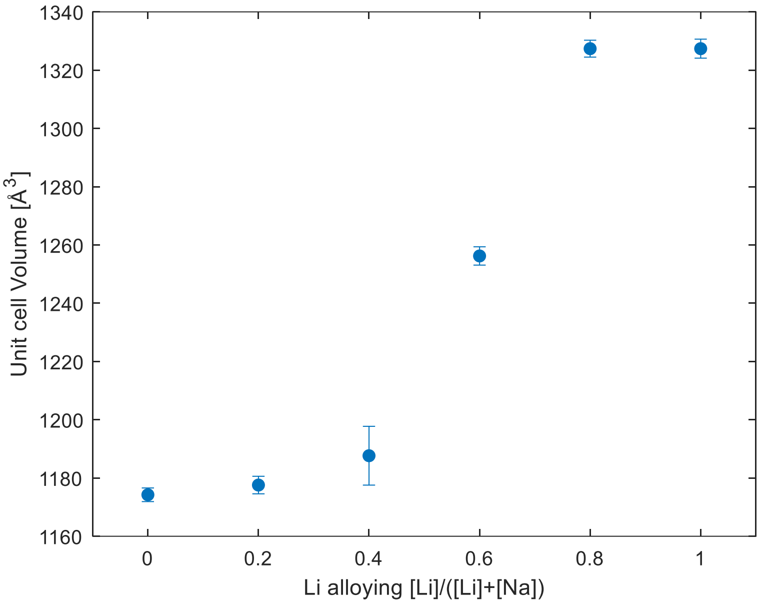

Figure S11. Calculated unit cell volume as a function of Li content for $Cs_2Na_{1-x}Li_xIn_{0.9}Sb_{0.1}Cl_6$ nanoparticles (x=0, 0.2, 0.4, 0.6, 0.8, 1).

References

(1) Hÿtch, M. J.; Snoeck, E.; Kilaas, R. Quantitative Measurement of Displacement and Strain Fields from HREM Micrographs. *Ultramicroscopy* **1998**, *74* (3), 131–146. https://doi.org/10.1016/S0304-3991(98)00035-7.

(2) Peters, J. Strain++. https://jjppeters.github.io/Strainpp/.

(3) Jing, Y.; Liu, Y.; Jiang, X.; Molokeev, M. S.; Lin, Z.; Xia, Z. Sb^{3+} Dopant and Halogen Substitution Triggered Highly Efficient and Tunable Emission in Lead-Free Metal Halide Single Crystals. *Chem. Mater.* **2020**, *32* (12), 5327–5334. https://doi.org/10.1021/acs.chemmater.0c01708.

(4) Kshirsagar, A. S.; Arfin, H.; Banerjee, S.; Mondal, B.; Nag, A. Colloidal Sb^{3+}-Doped $Cs_2InCl_5 \cdot H_2O$ Perovskite Nanocrystals with Temperature-Dependent Luminescence. *J. Phys. Chem. C* **2021**, *125* (50), 27671–27677. https://doi.org/10.1021/acs.jpcc.1c08720.

(5) Gong, Z.; Zheng, W.; Huang, P.; Cheng, X.; Zhang, W.; Zhang, M.; Han, S.; Chen, X. Highly Efficient Sb^{3+} Emitters in 0D Cesium Indium Chloride Nanocrystals with

Switchable Photoluminescence through Water-Triggered Structural Transformation. *Nano Today* **2022**, *44*, 101460. https://doi.org/10.1016/j.nantod.2022.101460.

(6) Noculak, A.; Morad, V.; McCall, K. M.; Yakunin, S.; Shynkarenko, Y.; Wörle, M.; Kovalenko, M. V. Bright Blue and Green Luminescence of Sb(III) in Double Perovskite Cs_2MInCl_6 (M = Na, K) Matrices. *Chem. Mater.* **2020**, *32* (12), 5118–5124. https://doi.org/10.1021/acs.chemmater.0c01004.

(7) Wojdyr, M. Fityk: A General-Purpose Peak Fitting Program. *J. Appl. Crystallogr.* **2010**, *43* (5), 1126–1128. https://doi.org/10.1107/S0021889810030499.
